# The full phase space dynamics of a magnetically levitated electromagnetic vibration harvester

**DOI:** 10.1038/s41598-021-95478-6

**Published:** 2021-08-16

**Authors:** Tobias Willemoes Jensen, Andrea R. Insinga, Johan Christian Ehlers, Rasmus Bjørk

**Affiliations:** Department of Energy Conversion and Storage, Technical University of Denmark - DTU, Anker Engelunds Vej 1, 2800 Kgs. Lyngby, Denmark

**Keywords:** Devices for energy harvesting, Magnetic properties and materials

## Abstract

We consider the motion of an electromagnetic vibrational energy harvester (EMVEH) as function of the initial position and velocity and show that this displays a classical chaotic dynamical behavior. The EMVEH considered consists of three coaxial cylindrical permanent magnets and two coaxial coils. The polarities of the three magnets are chosen in such a way that the central magnet floats, with its lateral motion being prevented by enclosion in a hollow plastic tube. The motion of the floating magnet, caused by e.g. environmental vibrations, induces a current in the coils allowing electrical energy to be harvested. We analyze the behavior of the system using a numerical model employing experimentally verified expressions of the force between the magnets and the damping force between the floating magnet and the coils. We map out the phase space of the motion of the system with and without gravity, and show that this displays a fractal-like behavior and that certain driving frequencies and initial conditions allow a large power to be harvested, and that more stable states than two exists. Finally, we show that at leasts fifth order polynomial approximation is necessary to approximate the magnet-magnet force and correctly predict the system behavior.

## Introduction

**Figure 1:**
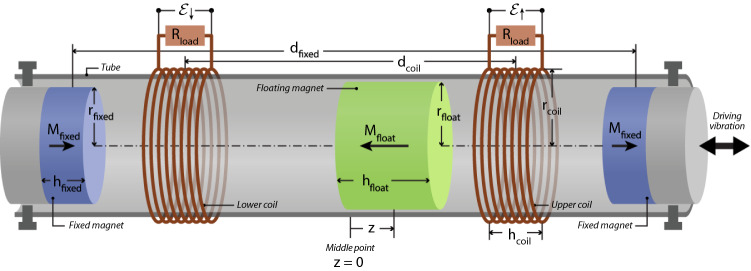
Schematic drawing of the considered EMVEH device. The two blue magnets in each end of the tube are fixed and have their magnetisation, $M_{{{\text{fixed}}}}$, pointing in the same direction. The green magnet in the middle is free to move and has its magnetisation, $M_{{{\text{float}}}}$, pointing in the opposite direction. Each of the two coils coloured in red is coupled to an external resistive load. The radius, *r*, and the height, *h*, of the different components are also indicated.

Remote microelectronic devices with a low power consumption have the potential to be supplied with energy harvested from the surrounding environment, saving expensive battery replacements for these devices. A direct sources of ambient energy is vibrations, where multiple concepts for harvesting this source of energy have been suggested^1,2^. Here we consider an electromagnetic vibration harvester (EMVEH), which generates power by vibrating a magnet past a set of coils. Specifically, we consider the EMVEH where a middle magnet is levitated by placing it between two coaxial cylindrical permanent magnets and enclosing it in a tube^2,3^, to prevent it from flipping. This middle magnet can then be vibrated through a set of coils, inducing an electromotive force. Such a design is illustrated in Fig. 1. The middle magnet experiences a non-linear restoring force and the system can be considered as a one-dimensional non-linear oscillator^3–5^. Such EMVEH harvesters prototypes are very simple to make, as all that is needed are off-the-shelf components such as permanent magnets, coils (typically available from audio equipment suppliers), acrylic tube and simple 3D-printed parts. Such harvesters can also be miniaturized although this involves more specialized production techniques^6^.

In the harvester the energy is extracted through coils placed around the tube, where the changing magnetic flux through the coils induces an electromotive force according to Faraday’s law. The magnetic field generated by the coils when they are excited by the induced electromotive force opposes the relative motion between the levitated magnet and the coils. Therefore, the effect of this interaction is to exert a damping force on the floating magnet.

The magnet-magnet restoring force, also termed the magneto-elastic force, present in the EMVEH is highly non-linear when the floating and fixed magnets are close to each other. A large number of mathematical expressions for both the magnet-magnet force and the induced electromotive force between the floating magnet and the coils have been derived^7^. However, the magnet-magnet force is often approximated by a linear and a cubic term, $k z + \beta z^3$, which makes the governing equation of motion resemble the Duffing equation^3,8–10^, although with a position-dependent dampening term. Such an approximation generally allows for an analytical solution for e.g. the produced power to be obtained^11^. However, the influence of this expression on the dynamics and stability of the system has not been studied in detail.

Furthermore, there is a large gap in knowledge with regards on how to harvest the maximum amount of power from a vibration source using an EMVEH. It has been shown that energy harvesters with a non-linear restoring force are superior compared to those with a linear one due to the wider range of frequencies that can excite the oscillator to a resonant state^12^. While reasonable power densities, with a maximum of 8 mW/cm$^3$^13^, has been demonstrated^7^, the non-linear force present in EMVEHs introduces frequency regions with multiple stable solutions, which creates situations where the harvester can jump between regions of low and high excitation^14,15^, with an order of magnitude difference in power production between these states. However, the driving frequency which causes these different states to appear is not a parameter that can be controlled for an EMVEH in a real-world application. Instead, the initial conditions in the EMVEH, i.e. the initial position and velocity of the floating magnet, can be controlled but their influence on the harvested power is not known.

Our hypothesis is that by understanding this influence, the harvested power and the frequency harvesting range can be optimized, which is needed for EMVEHs to be truly useful for a broad range of applications. To investigate this, we here present an analysis of an EMVEH system using a numerical model based on experimentally validated expressions for the magnet-magnet restoring force and the induced electromotive force^16^. We explore the full dynamics of the system to investigate how the initial conditions of the floating magnet influence the harvested power and compare this to the standard approach of approximating the magnet-magnet force with a polynomial expression. Further, we also explore the effect of gravity on the system.

A modeling approach to investigating the phase space of an EMVEH is warranted for three reasons. For the first, we use experimentally verified expressions for the magnet-magnet force and the dampening force from the coils, ensuring a fully realistic model. Secondly, a model allows us to ignore mechanical friction in the device, for which no general description exists that can be used to generalize the found results. Thirdly, using a modeling approach, we can conduct a large parameter space survey, which would be impossible using an experimental approach. In this work more than 7 million numerical experiments have been conducted, an impossible number to realize experimentally.

## Theory and modelling

In this study we model the dynamics of an EMVEH, with specific focus on the harvested power as function of the initial conditions of the harvester. In order to compute the harvested power, it is necessary to describe the dynamic behavior of the floating magnet which is governed by Newton’s second law. The model for this is described in the following sections.

The model presented in similar to many of the existing models presented in Ref.^7^ and includes the same physics as these. However, the novel part of this work is not a new model, but an exploration of the chaotic behavior that the model gives rise to, the number of steady operating states for an EMVEH harvester and the produced power in these states.

### The dynamical system

We will denote with *z*, ${\dot{z}}$, and $\ddot{z}$ the position, velocity and acceleration of the floating magnet, respectively. For now we will assume that the only moving part of the device is the floating magnet. Newton’s second law is then expressed as

1 $$m_{{{\text{float}}}} \ddot{z} = F_{e} (\dot{z},z) + F_{m} (z) + F_{g}$$

where $m_{{{\text{float}}}}$ is the mass of the floating magnet, $F_e$ is the force due to the interaction of the floating magnet with the coils, $F_m$ is the force due to the interaction of the floating magnet with the fixed magnets, and $F_g$ is the gravitational force. As shown in Eq. (1), the magnet-coil force $F_e$ depends on the position of the floating magnet and its velocity, the magnet-magnet force $F_m$ depends only on the position, and the gravitational force is constant: $F_g=-m g$, where g$=9.8$ m/s${}^2$ denotes the gravitational acceleration. As will be discussed in the "Mechanical friction" section, the effect of mechanical friction is not included. The expressions of the two terms $F_m$ and $F_e$ will be presented in the "Magnet-magnet force" section and the "Magnet-coil force" section, respectively. In the "Driving force" section we will introduce the effect of the external driving vibrations.

### Magnet-magnet force

In this section we consider the interaction between the floating magnet and the two fixed magnets. The force between two coaxial cylindrical uniformly-magnetized magnets has previously been obtained through a Fourier approach^16–19^. This approach consists of employing the Fourier transform to evaluate over the reciprocal space the integral of the dipole-dipole interaction energy density between the magnets using a specific shape function. The force between one of the fixed magnets and the floating magnet is directed along the *z*-axis and is given by^16^

2 $$F_{{12}} (z_{{12}} ) = 4\pi \mu _{0} M_{{{\text{float}}}} M_{{{\text{fixed}}}} r_{{{\text{float}}}} r_{{{\text{fixed}}}} \frac{{\partial J_{d} }}{{\partial z_{{12}} }}$$

Here $M_{{{\text{float}}}}$ and $M_{{{\text{fixed}}}}$ are the remanent magnetisations of the floating and fixed magnet respectively, $z_{12}$ is the relative distance between the floating and fixed magnet and $J_d(z_{12})$ is the dipolar coupling integral:

3 $$J_{d} (z_{{12}} ) = \int_{0}^{\infty } {\frac{{J_{1} (q)J_{1} \left( {q\frac{{r_{{{\text{fixed}}}} }}{{r_{{{\text{float}}}} }}} \right)}}{q}} \sinh (\tau _{{{\text{float}}}}q )\sinh \left({ \frac{{r_{{{\text{fixed}}}} }}{{r_{{{\text{float}}}} }}\tau _{{{\text{ fixed}}}}q } \right)e^{\frac{{ - z_{{12}} q}}{{r_{{{\text{float}}}} }}}dq$$

Here $J_1$ is the Bessel function of first kind, *q* is the reciprocal space variable, $r_{\text{float}}$ and $r_{\text{fixed}}$ are the radii of the magnets, and $\tau _{\text{float}}$ and $\tau _{\text{fixed}}$ are the height-to-radius ratios of the magnets, $\tau =h/r$. This force expression is similar to other expressions presented in Ref.^7^.

As shown in Fig. 1, we here consider a system with two fixed magnets. The relative orientation between the floating magnet and each of the fixed magnets is such that the mutual force is repulsive in both cases. Therefore, the combined effect of the two interactions is to create a non-linear restoring force acting on the middle magnet. Its stable equilibrium position is thus the middle point between the two fixed magnets, corresponding to $z=0$. Since the two fixed magnets are separated by a distance $d_{\text{fixed}}$, the relative distances between the floating magnet and each of the fixed magnets are $z_{12}=d_{\text{fixed}}/2\pm z$, respectively. In conclusion, the net magneto-elastic or magnet-magnet force acting on the floating magnet is the sum of the two terms:

4 $$F_{m} (z) = F_{{12}} (d_{{{\text{fixed}}}} /2 + z) - F_{{12}} (d_{{{\text{fixed}}}} /2 - z)$$

The reason for the minus sign in the second term on the right-hand side of the previous equation is that the signs of the force exerted by the two fixed magnets is opposite. Clearly, the magneto-elastic force is conservative since it is the derivative of the associated magnetostatic potential energy.

We have implicitly assumed that the magnetization is uniform within each magnet and independent of the magnetic field experienced by the magnets. However, we emphasize that this procedure for computing the force expression has been experimentally validated^16^, and that these two assumptions have been verified to be realistic enough for the system under consideration.

### Magnet-coil force

In this section we consider the interaction between the floating magnet and the coils. As shown in Fig. 1 we consider two *n*-turns coils belonging to two different electrical circuits, as this will result in the maximum induced power. We will initially focus on one turn of one of the two coils (e.g. the $i$th turn of the upper coil). The considerations that are valid for the upper coil can be analogously applied to the identical lower coil. We will denote with $z_{i}$ the relative distance between the floating magnet and the $i$th turn. Since all the turns of both the coils are fixed, the relative velocities between the floating magnet and all the turns are the same, and will be denoted by ${\dot{z}}= \frac{dz_i}{dt}$, $\forall i$.

When the floating magnet moves towards or away from one of the coils, the magnetic field generated by the magnet induces an electromotive force ${\mathcal {E}}_i$ in the coil’s turn. The electromotive force causes a current *I* which gives rise to a magnetic field generated by the coil. The magnetic field from the coil exerts a force on the magnet which oppose the relative motion between them. We will denote with $F_{i}$ the force exerted by one coil’s turn on the floating magnet.

From an energy perspective, the instantaneous power $P_{i}^{(c)}$ induced in the turn, which of course is the purpose of the device, is equal to the associated rate of decrease $P_{i}^{(m)}$ in the kinetic energy of the floating magnet. Therefore, the effect of this interaction on the movement of the floating magnet is equivalent to that of a damping force in the sense that it always reduces its velocity relatively to the coil.

The excitation power of the coil’s turn is equal to the product between the current and the electromotive force: $P_{i}^{(c)}= {\mathcal {E}}_i I$, where it is noted that the current *I* is the same for all the turns of the same coil since they are connected in series. We denote by $R=R_{\text{coil}}+R_{\text{load}}$ the total resistance of each of the two coils’ circuits, i.e. the inner resistance of one coil plus the corresponding external load. As we discuss at the end of this section, in this work we follow previous publications^20–24^ in neglecting the self-inductance of the coils’ circuit and the mutual inductance between the two coils. Therefore, the current is equal to $I = {\mathcal {E}}/R$, where the total electromotive force is the sum of the individual contributions from all the turns: ${\mathcal {E}} = \sum _i^n {\mathcal {E}}_i$. The power subtracted from the magnet’s kinetic energy by the interaction with one turn is given by the product of the corresponding force $F_{i}$ and the velocity of the magnet: $P_{i}^{(m)} = -F_{i} {\dot{z}}$. We can thus express the energy conservation law for this interaction as:

5 $$\begin{aligned} P_{i}^{(c)} = {\mathcal {E}}_i I = {\mathcal {E}}_i \frac{{\mathcal {E}}}{R} =- F_{i} {\dot{z}} = P_{i}^{(m)} \end{aligned}$$

Solving the previous equation with respect to the force we obtain:

6 $$\begin{aligned} F_{i} = - {\mathcal {E}}_i \frac{{\mathcal {E}}}{R{\dot{z}}} \end{aligned}$$

The electromotive force can be found from Faraday’s law of induction^25^:

7 $$\begin{aligned} {\mathcal {E}}_i = -\frac{d\phi _i}{dt} = -\frac{dz_i}{dt}\frac{d\phi _i}{dz_{i}} = -{\dot{z}}\frac{d\phi _i}{dz_{i}} \end{aligned}$$

where $\phi _i=\phi _i(z_{i})$ is the flux through the $i$th coil’s turn, which is a function of the relative distance $z_{i}$ between the magnet and the turn. Plugging Eq. (7) into Eq. (6) for both the single turn and for ${\mathcal {E}}$ we obtain:

8 $$\begin{aligned} F_{i}({\dot{z}},z_{i}) = -{\dot{z}} \frac{1}{R} \frac{d\phi _i}{dz_{i}} \sum _{j=1}^n \frac{d\phi _j}{dz_{j}} \end{aligned}$$

The total force acting on the floating magnet is obtained by adding the individual contributions from all the turns of each coil. Denoting by $\zeta _i$ the position of the $i$th turn, the relative distance between the floating magnet and the turn is written as: $z_i = \zeta _i-z$. The total force from one of the coils (e.g. the upper coil) is thus given by:

9 $$\begin{aligned} F_e^{\uparrow }({\dot{z}},z) = \sum _{i=1}^n F_{i}({\dot{z}},\zeta _i-z) \end{aligned}$$

Thus the damping force is a function of both position and velocity of the floating magnet relative to the coil. In particular, the force is proportional to the velocity of the floating magnet: $F^{\uparrow }_e({\dot{z}},z) = -{\dot{z}} c^{\uparrow }(z)$. The proportionality factor $c^{\uparrow }$, which can thus be thought of as a damping coefficient, depends on the position of the floating magnet according to:

10 $$\begin{aligned} c^{\uparrow }(z) = \frac{1}{R} \left( \sum _{i=1}^n \left. \frac{d\phi _i}{dz_{i}} \right| _{z_i = \zeta _i-z}\right) ^2 \end{aligned}$$

In order to determine the actual damping force, the derivative of the flux through each coil’s turn must be determined. By using again the principle of Fourier transform, an experimentally verified analytical expression for the flux through a single turn of a coil (i.e. a ring) can be obtained^16^. It should be noted that two different expressions appear depending on whether the magnet is inside or outside the ring, where in the former case, the magnetisation, **M**, contributes to the flux, $\phi$, in addition to the demagnetising field **H**. The analytical expressions for the flux derivative $\frac{d\phi }{dz_{i}}$ from a single-turn coil are^16^:

11a $$\left( {\frac{{d\phi }}{{dz_{i} }}} \right)_{{{\text{in}}}} {\text{ = }}2\pi \mu _{0} M_{{{\text{float}}}} r_{{{\text{coil}}}} \int_{0}^{\infty } {J_{1} } (q)J_{1} \left( {q\frac{{r_{{{\text{coil}}}} }}{{r_{{{\text{float}}}} }}} \right)\sinh \left( {\frac{{qz_{i} }}{{r_{{{\text{float}}}} }}} \right)e^{{ - q\tau _{{{\text{float}}}} }} dq{\text{ }}$$

11b $$\left( {\frac{{d\phi }}{{dz_{i} }}} \right)_{{{\text{out}}}} = 2\pi \mu _{0} M_{{{\text{float}}}} r_{{{\text{coil}}}} \int_{0}^{\infty } {J_{1} } (q)J_{1} \left( {q\frac{{r_{{{\text{coil}}}} }}{{r_{{{\text{float}}}} }}} \right)\sinh (q\tau _{{{\text{float}}}} )e^{{\frac{{ - qz_{i} }}{{r_{{{\text{float}}}} }}}} dq$$

where $r_{\text{coil}}$ is the radius of the coil.

As shown in Fig. 1, the prototype under consideration includes two identical *n*-turns coils symmetrically placed around the point $z=0$. Denoting by $d_{\text{coil}}$ the distance between the midpoints of two coils, by $h_{\text{coil}}$ the height of the coils, and by $\delta =h_{\text{coil}}/(n-1)$ the distance between turns, the position of the $i$th turn of the upper coil is $\zeta _{i\uparrow } = d_{\text{coil}}/2-h_{\text{coil}}/2+(i-1)\delta$, for $i=1,\dots ,n$. Similarly, the position of the $i$th turn of the lower coil is $\zeta _{i\downarrow } = - \zeta _{i\uparrow }$. The total force acting on the floating magnet is simply the sum of the forces from each coil:

12 $$\begin{aligned} F_e({\dot{z}},z) = F_e^{\uparrow }({\dot{z}},z) + F_e^{\downarrow }({\dot{z}},z) \end{aligned}$$

As for the magnet-magnet force, some approximations have been assumed in order to derive the magnet-coil force expression. In particular, the thickness of the wire is neglected, and all the turns are assumed to have the same radius $r_{\text{coil}}$, while in reality a coil typically have a few radial layers. Moreover, the coil is modelled as a series of parallel circles rather than a helix. Finally, as in previous works^21–24^, here we only consider the inductive coupling between the floating magnet and all the coils, thus neglecting the self-inductance *L* of each of the coils’ circuit and the mutual inductance $L_{\text{mutual}}$ between them. In order to include self-inductance effects, the current in each coil would have to be considered as an additional degree of freedom governed by a separate equation coupled with Eq. (1).

13a $$\begin{aligned}&L{\dot{I}}^{\downarrow } + L_{\text{mutual}} {\dot{I}}^{\uparrow }+ RI^{\downarrow } + {\dot{z}}\left( \sum _{i=1}^n \frac{d\phi ^{\downarrow }_i}{dz_{i}}\right) =0 \end{aligned}$$

13b $$\begin{aligned}&L{\dot{I}}^{\uparrow } + L_{\text{mutual}} {\dot{I}}^{\downarrow }+ RI^{\uparrow } + {\dot{z}}\left( \sum _{i=1}^n \frac{d\phi ^{\uparrow }_i}{dz_{i}}\right) =0 \end{aligned}$$

with

14 $$\begin{aligned} F_e({\dot{z}},z) = -I^{\uparrow } \left( \sum _{i=1}^n \frac{d\phi ^{\uparrow }_i}{dz_{i}}\right) - I^{\downarrow } \left( \sum _{i=1}^n \frac{d\phi ^{\downarrow }_i}{dz_{i}}\right) \end{aligned}$$

We recover our governing equation in the limit $L\rightarrow 0$, $L_{\text{mutual}}\rightarrow 0$.

As for the magnet–magnet interaction, we emphasize that also the magnet-coil force expression has been experimentally validated^16^.

The load resistance connected to each of the two coils must be chosen so that the maximum harvested power is achieved. To do so, the load resistance has to be equal to the internal resistance of the coil (ignoring the coil self-inductance)^26^: $R_{\text{load}} = R_{\text{coil}}$. The modelled coils are based on the properties of a copper coil with $n=16$ turns, a wire radius of $r_ {\text{wire}} =0.13$ mm, leading to a resistance given by:

15 $$R_{{{\text{coil}}}} = 2\pi nr_{{{\text{coil}}}}\rho _{{{\text{copper}} }} /(\pi r_{{{\text{wire}}}}^{2} ) = 0.317\;\Omega$$

where $\rho _{\text{copper}} =1.68 \times 10^{-8}$ ${\Omega m}$ denotes the resistivity of copper. Therefore, the total resistance of each of the two circuits is $R = 0.634$ ${\Omega }$. The geometrical parameters are also listed in Tab. 1.

### Driving force

Of course the purpose of the EMVEH device is to harvest energy from environmental vibrations. The model discussed so far does not include the coupling with environmental vibrations. In this section we introduce the coupling of the device with the external vibrations.

Traditionally, an oscillatory driving force of prescribed amplitude is included in Eq. (1) as an additional term which directly affects the dynamics of the floating magnet. However, in reality the vibrations act on the external part of the device and not directly on the floating magnet. Here we follow a more realistic approach^4^, where the EMVEH is considered to be small relative to the vibrating structure to which it will be mounted. This makes it appropriate to describe the EMVEH device by a driving movement, as the fixed parts of the device (the coils and fixed magnets) are expected to completely follow the motion of the structure to which the device is attached. This means that the fixed magnets and coils all move with a fixed time-dependence, here termed $Z_{\text{drive}}(t)$. This means that the input arguments of the functions $F_m(z)$ and $F_e({\dot{z}},z)$ will be replaced according to^27^:

16a $$\begin{aligned}&z \rightarrow z-Z_{\text{drive}} \end{aligned}$$

16b $$\dot{z} \to \dot{z} - \dot{Z}_{{{\text{drive}}}}$$

We assume here that the driving motion is given by

17a $$Z_{{{\text{drive}}}} (t) = A\sin (\omega t)$$

17b $$\dot{Z}_{{{\text{drive}}}} (t) = - A\omega \cos (\omega t)$$

where *A* is the amplitude of the movement and $\omega =2\pi f$ is the angular frequency.

The final full equation governing the motion of the system is thus:

18 $$\ddot{z} = {\text{ }}\frac{1}{{m_{{{\text{float}}}} }}F_{e} \left( {\dot{z} - \dot{Z}_{{{\text{drive}}}} ,z - Z_{{{\text{drive}}}} } \right) + \frac{1}{{m_{{{\text{float}}}} }}F_{m} \left( {z - Z_{{{\text{drive}}}} } \right) - g$$

While Eq. (18) is used to mechanically evolve the system forward in time, the power harvested from the system is computed at every time step using Eq. (5) and thus there is a full coupling between the mechanical motion of the system and its electrical behavior.

The above equation is assumed to be non-stiff and is solved numerically using MATLAB’s ode45 with default tolerances of $\text {abstol}=10^{-3}$ and $\text {reltol}=10^{-6}$. The computed solution is sampled with 500 points for each driving-period.

As stated above the model presented here has experimentally validated expressions for all interaction terms in the governing equation of motion. The resulting differential equation is then numerically integrated using a standard ODE approach. The numerical solution has been verified to precisely satisfy the equation of motion given as input, i.e. the ODE solution does not change with changing tolerance, and thus the resulting solution is fully validated.

### Mechanical friction

Besides the effect of electromagnetic damping, studies of EMVEH devices may include mechanical friction between the floating magnet and the encasing tube and viscous friction experienced by the floating magnet as it moves through the air in the tube.

There are different models for mechanical friction forces between sliding surfaces^28^. The Coulomb model is the simplest, with most other models being improvements of this. In the Coulomb model, the friction force is proportional to the normal force between the surfaces in contact with the proportionality factor being the friction coefficient. In a vertical setup, the only lateral force pressing the floating magnet against the surface is of magnetic origin. However, for perfectly cylindrical and coaxial magnets this force is zero. Therefore this force is only due to imperfections such as a small lateral displacement of the floating magnet, for which a full three-dimensional model would be necessary to predict this friction coefficient.

In fact, in all the studies of EMVEH devices that include mechanical friction, the force is parameterized by determining one or more coefficient by means of fitting the simulation results with experimental measurements^29,30^. However, this fitting procedure cannot be applied unless experimental data is already available. The main purpose of our study is to propose a model that can be used during the design optimization step and to explore the chaotic behavior of a EMVEH system. Therefore, fitting with experimental data is not a viable option at this stage and therefore mechanical friction is ignored in the following, as has also been the case in other studied EMVEH systems^20–22,24^.

## Studied system

We consider the EMVEH attached to a structure that is much larger than itself. We assume that the structure vibrates as a sine with a fixed amplitude *A* of 0.5 cm and with a frequency *f* in the range of 0.1–20 Hz. The maximum acceleration of the driving system thus ranges from 0.003 to 4.3 g.

We consider a EMVEH system with the specific parameters as given in table 1 unless otherwise noted. The parameters are chosen realistically, allowing a subsequent prototype to be constructed. In this work, we have chosen to study a single EMVEH system, to fully explore the dynamics of this system.

**Table 1:** A table of the parameters for the considered system.

Parameter		Value	
Floating magnet	Height, $h_{\text{float}}$	1.6	cm
	Radius, $r_{\text{float}}$	1.5	cm
	Mass, $m_{\text{float}}$	22	g
	Magnetisation, $M_{\text{float}}$	$6.43\cdot 10^5$	A m
Fixed magnets	Distance, center to center, $d_{\text{fixed}}$	10.4	cm
	Height, $h_{\text{fixed}}$	0.5	cm
	Radius, $r_{\text{fixed}}$	1.5	cm
	Magnetisation, $M_{\text{fixed}}$	$6.84\cdot 10^5$	A m
Coils	Distance, center to center, $d_{\text{coil}}$	2.5	cm
	Height, $h_{\text{coil}}$	0.5	cm
	Radius, $r_{\text{coil}}$	1.0	cm
	Turns pr coil, *n*	16	
	Resistance pr coil, $R_{\text{coil}}$	0.317	$\Omega$
Driving system	Amplitude, *A*	0.5	cm
	Frequency, *f*	0.1–20	Hz
External	Load resistance, $R_{\text{load}}$	0.3173	$\Omega$

We consider the EMVEH mounted both horizontally and vertically. For the vertical system, i.e. when gravity is included, the equilibrium position of the floating magnet is shifted from $z = 0$ to $z_{eq} \approx -1.2$ cm, which means that the coils are no longer placed symmetric around the equilibrium position of the floating magnet.

Regarding the choice of coils for the studied system, we wish to note that the chosen coils have not been investigated to be the optimal coils for e.g. maximizing the harvested power. An additional parameter study would have to be conducted to choose the optimal coil and the optimal coil position, in order to harvest the maximum amount of power from the ambient vibrations. However, that is not the purpose of this manuscript. Here, our purpose is to choose “a” realistic coil, that allows power to be harvested and the dynamics of the system to be explored. As discussed previously, the load resistance is chosen to be equal to the internal resistance of the coils in order to maximize the harvested power^26^. In the following we give the total induced power in the coils. The power that can be harvested is equal to half of this value, as the load resistance is equal to the coil resistance.

**Figure 2:**
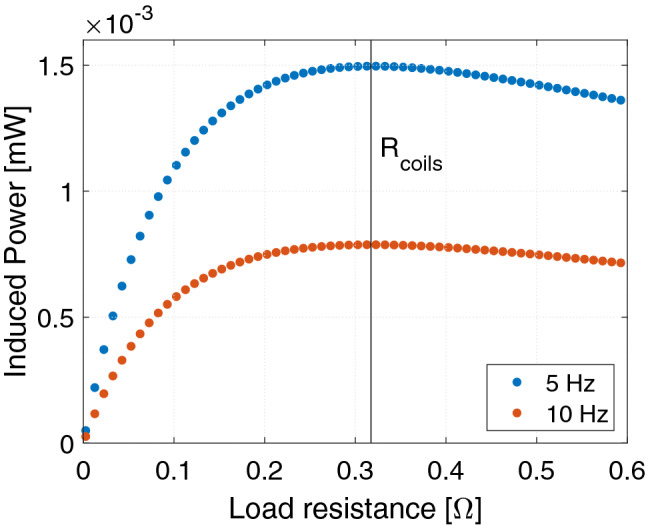
The induced power as function of the load resistance for the studied system starting from rest and a driving frequency of either 5 or 10 Hz for a horizontal system. The resistance of the coils have been indicated.

To show that the load resistance has to be equal to the value of the coil resistance, we in Fig. 2 show the induced power for the studied system, with the floating magnet started from a position of rest in the center of the tube, at 5 and 10 Hz respectively. As can clearly be seen from the figure, the induced power is maximized when the load resistance is equal to the coil resistance.

### Convergence to a steady state

The dynamic behavior of the EMVEH starting from a set of initial conditions will consist of a transient state and a steady state. It is only in the latter state that the amplitude of the oscillations and the energy extracted through the coils is of interest.

Here we search for periodicity in the solution *z*(*t*) over a wide range of driving frequencies $f= 1/T$, where *T* denotes the duration of a cycle of the driving oscillations. We will use the term *cycle* to refer to *periods in the driving movement*, as opposed to *periods in the solution* which still is referred to as *periods*. In fact, the duration of a period of the steady state solution is not necessarily equal to *T*, but might also span multiple cycles^31^:

19 $$\begin{aligned} z(t+nT) = z(t), \quad \forall t \end{aligned}$$

**Figure 3:**
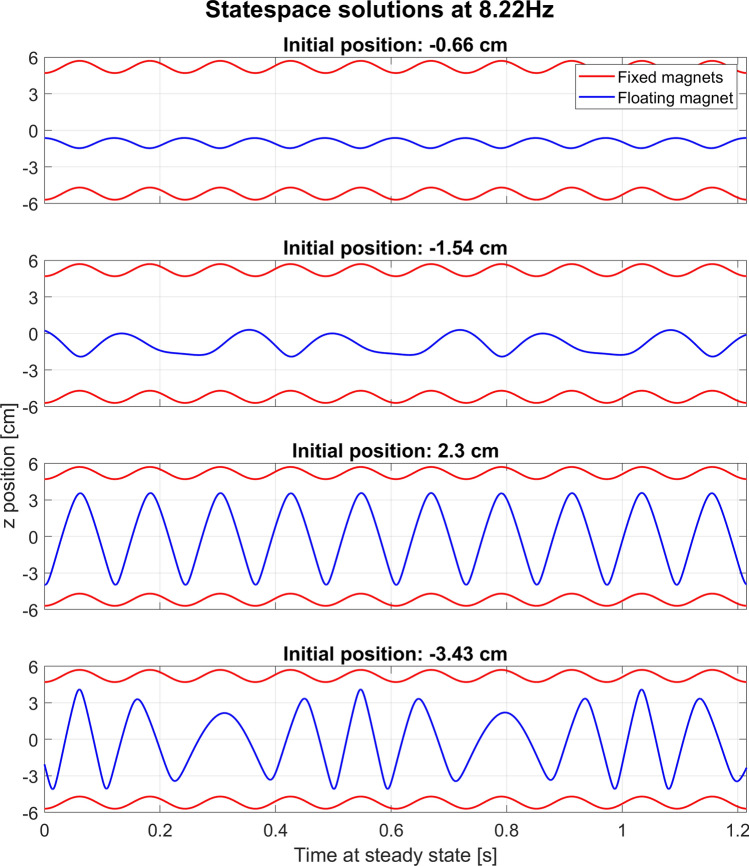
An illustration of the steady state behavior of the system with gravity at the same frequency of 8.22 Hz but different starting positions of the floating magnet as stated on the graphs. The initial velocity is − 7.09 cm/s in all cases. The movement is shown in a reference frame at rest, thus the fixed magnets also move.

In Fig. 3 we illustrate several solutions for the same frequency, but for different initial conditions of the floating magnet. As can be noticed from the figure, in some cases the periodicity can be greater than one cycle.

In every simulation the number of cycles needed for the solution to be periodic is determined by comparing the solution for the last *n* cycles to the previous *n* cycles. If the mean difference over these *n* cycles for some value of *n* is below 0.1%, this number of cycles is chosen as representative for a steady state solution since it has repeated itself. When the tolerance has been met and the simulation is terminated, the amplitude within the converged number of cycles is computed together with the mean extracted power in this range as computed using Eq. (5). If the tolerance is not met within the maximum number of cycles the solution is not accepted since the system did not converge to the steady state regime.

The maximum number of cycles compared, the number of time steps per cycle and the maximum number of cycles computed vary between the different system behaviors explored. The specific values are given in the sections below.

## Results

All data presented in the following sections, as well as all scripts for making the figures etc. are directly available from Ref.^32^.

### Hysteresis behavior

There are multiple ways to explore the state space of solutions of the system. One way is to explore the response of the system, when the driving frequency in gradually increased or decreased slowly enough for the system to relax to the steady state regime. In other words, the steady state solution calculated for a given frequency is used as initial conditions for the new frequency and the system is initially started from an initial position of zero and an initial velocity of zero. There is thus only one initial condition to the whole set of frequencies considered, contrary to the behavior explored in subsequent sections. Investigating the behavior of EMVEHs is traditionally done in this kind of experiment^3^.

**Figure 4:**
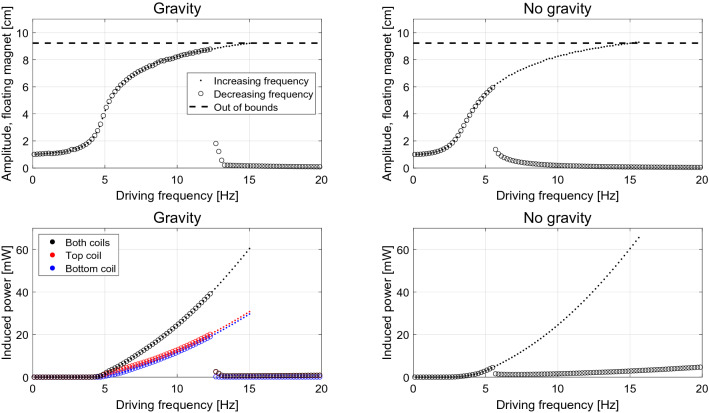
The frequency response of the system in terms of amplitude and induced power, with and without gravity. Note that the amplitude is that as seen from a rest frame. The dots shows the system as it starts at rest, and then slowly ramp up the frequency, giving time at each step for steady state to settle. The circles shows the reverse, where the system starts at 20 Hz, and then slowly ramps down. Only data for systems that reached steady state is shown.

Here we consider a maximum number of cycles compared to be 200, and the calculation is stopped after 2000 cycles. The number of time steps per cycle is 1000. Performing this analysis on the system considered here, we calculate the amplitudes of the floating magnet and induced power as shown in Fig. 4, where points are from low to high frequencies, and circles are reverse. Note that it is the absolute amplitude of the floating magnet oscillation as seen from a rest frame that is shown, i.e. the amplitude is not relative to the moving system.

Opposed to a linear equation, this system does not have a clearly defined resonance frequency. This phenomenon is sometimes called a *foldover effect*, since the bell-shaped peak normally seem for a linear oscillator folds over^15^, thus exhibiting multiple solutions. As shown in Fig. 4, as the driving frequency is increased the amplitude of the floating magnet initially increases quickly before reaching a plateau, for both systems with and without gravity. For driving frequencies greater than 15 Hz the oscillating amplitude of the free magnet is larger than the distance between the fixed magnets. In reality, under these conditions the driving oscillation would cause the floating magnet to physically hit the fixed magnets. These results are thus unphysical and will be disregarded.

When the driving frequency is slowly reduced from 20 Hz, the system does not immediately return on the same set of steady-state solutions that were found for increasing frequency. Instead, the solutions initially follow a different curve characterized by smaller amplitude of oscillations. This hysteresis behavior, i.e. that the state of the system depend not only on the current external conditions but also on its history, is well-known for EMVEHs^3,24,30^, confirming the model presented here. It is important to note that there are two known states of the system: a high power and a low power state, and the system transitions between these at a fixed frequency. It is also worth noting that for the case with gravity the hysteresis region is smaller than for the case with no gravity, and in the latter case it is almost impossible to produce any power on the decreasing frequency branch.

From the point of view of bifurcation theory, this kind of behavior is known as the previously described foldover effect, which is also exhibited by the much simpler Duffing equation. Foldover is characterized by two bifurcation points which delimit the region where multiple equilibrium solutions are possible. Outside of this interval of the driving frequency axis only one stable solution exist. Inside the interval we observe two stable solutions, and the theory predicts^15,31^ the existence of an additional unstable solution within this interval, which our convergence criteria prevents us from observing. These are the features of two supercritical pitchfork bifurcations^31^.

Moreover as discussed in the "Convergence to a steady state" section, we observe periodic solutions with different periods, which is the signature of period-doubling bifurcations. Finally, in certain regimes chaotic behavior can be observed, as will be discussed subsequently. All these kinds of bifurcations are present for the case of the Duffing equation^31^, and are therefore expected also for the dynamical system considered in this work which can be thought of as a similar but more general case of the Duffing equation.

### Steady state solution dependence on the initial conditions

The full state space of the system is explored by calculating the steady-state solution as function of the starting position and velocity of the floating magnet. We have computed the amplitude of the resulting oscillation and the induced power for 128 different initial velocities ranging from − 100 to 100 cm/s, and 128 different initial positions ranging from − 4 to 4 cm. The range of initial positions were chosen to be slightly smaller than the distance between the two fixed magnets. The number of cycles compared to find the steady state solution is 50, and the calculation is stopped after 1000 cycles. The number of time steps per cycle is 500. The reduced numbers with respect to the values used in Sec. 4.1 are to decrease the computation time of the model.

**Figure 5:**
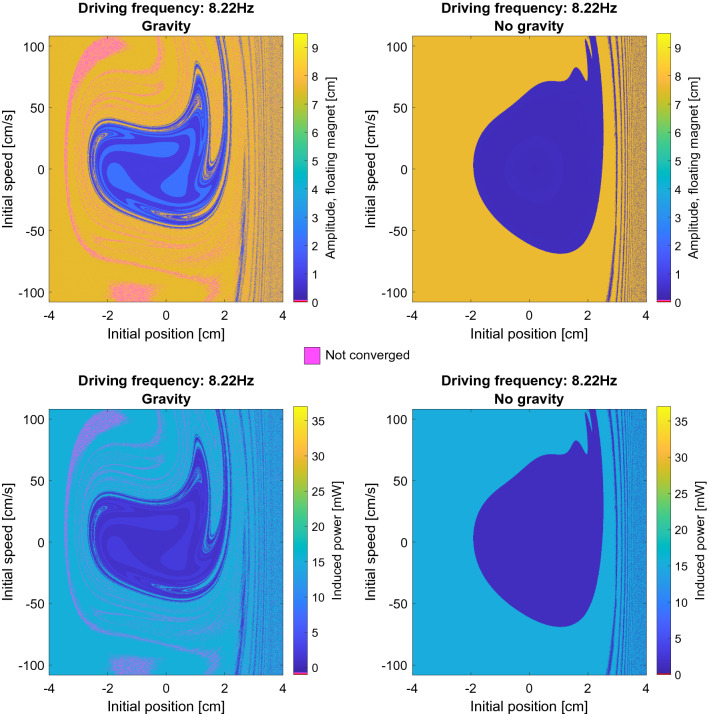
The amplitude of the floating magnet for 8.22 Hz with and without gravity for a range of initial positions and initial speeds and b) the corresponding induced power in the coils. Note that the color pink, which does not appear in the color scale, corresponds to systems that have not converged to a steady state solution.

We consider the range of driving frequencies from 0.1 to 20 Hz in 300 steps. The resulting state space plot, shown in a video with increasing frequency, is available as part of the supplementary material for this article. In Fig. 5 the state space plot of the resulting amplitude of the floating magnet is shown for a fixed driving frequency of 8.22 Hz in a higher resolution, 1800 different initial velocities ranging from − 108 to 108 cm/s, and 1000 different initial positions ranging from − 4 cm to 4 cm.

**Figure 6:**
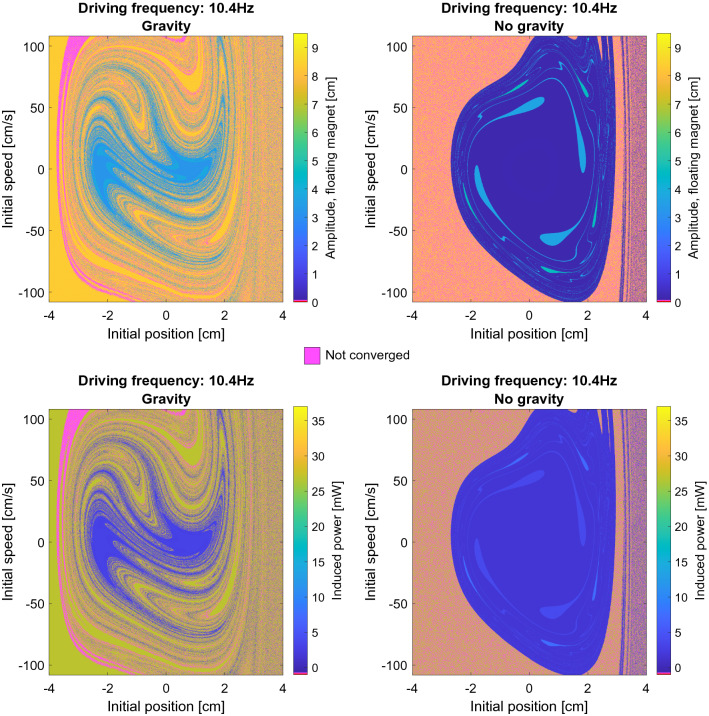
The amplitude of the floating magnet for 10.4 Hz with and without gravity for a range of initial positions and initial speeds and b) the corresponding induced power in the coils. Note that the color pink, which does not appear in the color scale, corresponds to systems that have not converged to a steady state solution.

As can be seen from the figure, due to the non-linearity of the equation of motion the state space plot exhibits features that are typical for chaotic dynamical systems^33^. In particular, the boundary between regions leading to different steady-state solutions may be fractal. In these regimes, a small change in the initial conditions may correspond to a strikingly different steady state behavior. The phase diagram for this system displays significantly more features and a more fractal-like nature compared to the previously computed phase diagrams for the Duffing equation^15,33^. Figure 6 shows the state space plot for a different driving frequency, i.e. 10.4 Hz. The chaotic structure is even more evident for this choice of frequency.

When gravity is not included, i.e. right panels in Figs. 5 and 6, the basin of attraction corresponding to different steady state solutions are less finely intertwined than they are when gravity is included. This is likely due to the coils being asymmetrical placed with respect to the equilibrium position of floating magnet when gravity is included.

What should also be observed from the state space plots is that for most of the frequencies shown, more than two steady state solutions exists. This means that more states than the classically known “high power” and “low power” states from frequency response experiments, as shown in Fig. 4, exists. It is interesting to explore the properties of these additional states, which the system does not access in the traditional frequency response experiments.

**Figure 7:**
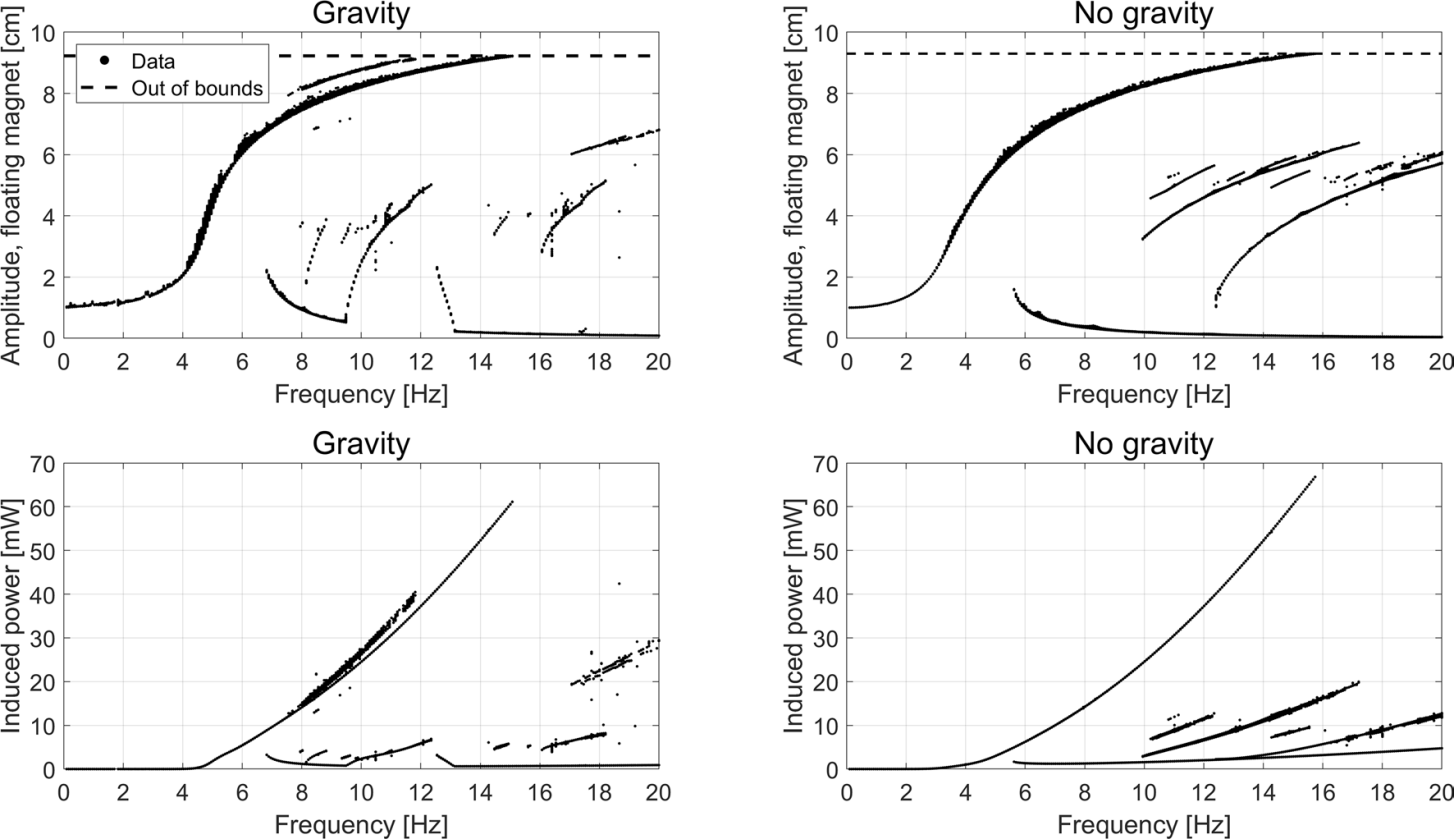
The amplitude and the induced power for 32 x 32 different initial conditions in position and velocity, as described in the text, with and without gravity. Only data for systems that reached steady state is shown.

The properties of these states can be investigated by plotting the amplitude of the floating magnet and the power induced as function of the frequency for all initial conditions considered. This is shown in Fig. 7 for 32 different initial velocities ranging from − 100 to 100 cm/s, and 32 different initial positions ranging from − 4 to 4 cm. The number of initial conditions have been reduced from 128 × 128 to 32 × 32 in order not to clutter the figure. Visualizing the state space in this way, one can see that compared to Fig. 4 at higher frequencies than 7 Hz there are multiple steady states that cannot be found through a simple ramping of the driving frequency. In other words only a small part of the phase space shown in Figs. 5 and 6 are explored in Fig. 4, while Fig. 7 shows this full phase space. These previously unreachable steady state solutions show vastly different amplitudes and corresponding induced powers. Note for example around 6–8 Hz there are two branches with vastly different amplitudes of steady states possible. In the “lower” branch the steady state amplitude is around 2 cm—a situation which results in almost no energy harvested, while in the “upper” branch the amplitude is around 7 cm, resulting in a much larger harvested power. It is also worth noting that for the system with gravity, a higher induced power state than the traditional high power state is possible between 8 and 12 Hz, with a gain in induced power around 5%.

### Polynomial approximation of the magnet force

In literature the restoring magnetic force is often approximated by a polynomial expression^3,7–9^. In this section we investigate the consequences of this approximation for the system considered here, by calculating the amplitude and induced power both for the true force and for various polynomial approximations fitted to the true force.

**Figure 8:**
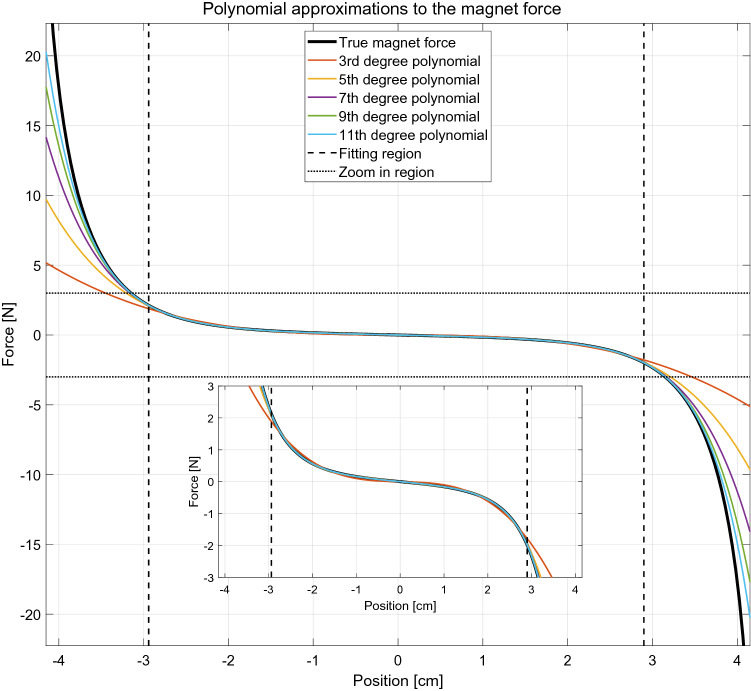
Polynomial fits of increasing order of the true force, $F_m$.

When fitting a polynomial to the true magnet force, we consider only the middle region of the system, as indicated in Fig. 8 by the vertical dashed lines. This restriction is applied because when fitting for the whole interval the result is dominated by the exponential ends of the true force curve, which leads to an unphysical negative slope of the polynomial approximation around the middle-point of the curve. The chosen range of fitting is the largest range for which the resulting 3rd degree polynomial is monotonic.

In Fig. 8 we show the polynomials of increasing order which best fit the true magnetic force in the interval discussed above. It should be emphasised that the result shown in Fig. 8 corresponds to the specific system considered. For system with other dimensions, the exponential-like behavior of the true force can mean that especially polynomials of low degree cannot accurately approximate the true force curve. Note that in a Duffing-like equation the conservative force would be expressed as $\alpha {}x+\beta {}x^3$, i.e. as the 3rd-order polynomia considered here.

**Figure 9:**
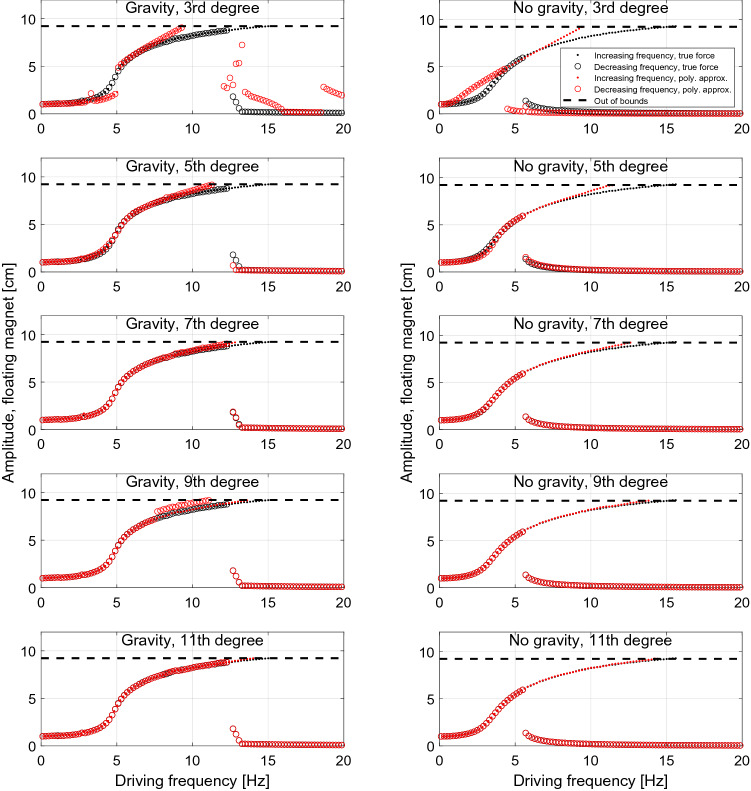
The frequency response from different polynomial approximation of the true force, $F_m$, with and without gravity.

In Fig. 9 the frequency response of the system compared to the true force, $F_m$, is shown. The frequency response is computed in the same manner as in Section “Hysteresis behavior”, i.e. increasing or decreasing the driving frequency by small steps and letting the system relax to the steady state regime before proceeding to the following frequency step. The number of cycles considered are the same as in Secton “Hysteresis behavior”.

From a practical point of view these results show that even using a relatively low order polynomial, 5th degree and higher, the overall dynamical behaviour of the system is captured and the discontinuous jump is correctly predicted, as a previous investigation have also shown^23^. Note that the Duffing-type equation does not accurately predict the behavior of the EMVEH. Of course this conclusion is only true for the specific system studied and it should be remarked that e.g. if the fixed magnets are moved much further apart the force curve will be harder to approximate with a polynomial function, leading to inaccurately calculated values. It is also seen that the results computed with the polynomial approximations are more accurate at lower frequencies since the resulting oscillation amplitude is smaller for this frequency range.

### Discussion

As highlighted by our investigation, it is important to include the effect of gravity since the shift of the equilibrium position from the middle point breaks the symmetry, and this may lead to radically different dynamics. Thus the same harvester operated in vertical and horizontal position will provide quite different power.

As discussed in the "Magnet-coil force" and "Mechanical friction" sections in this work we did not consider the effect of the mechanical friction on the behavior of the system, nor the self- and mutual inductances of the coils. Including both of these effect and studying their impact on the state space of the system is of interest in a future study. Regarding experimental validation of the results, it would be extremely prohibitive to conduct the large number of simulations performed in this study. For example in Figs. 5 and 6 alone more than 7.2 million numerical experiments were conducted, which would be impossible to replicate experimentally. However, selected experiments at the different phase regions discovered could be performed to experimentally confirm the modelling results.

Another important point to consider is the stability of the steady state solutions. As shown in Fig. 5 and Fig. 6 in certain situations the system is extremely sensitive to small changes in the initial conditions. In this regime, a small perturbation, which is not unlikely under real operating conditions, might cause the system to undergo a transition to a drastically different behavior. Such transitions could have a negative effect such as a reduction of the harvested power or even damage to the device resulting from collisions between the magnets. There are several possible strategies to avoid such faults in the system. The most straightforward approach is to operate the device in conditions that are not too sensitive to small perturbations. These correspond to the most homogeneous regions of the state space plots shown in Figs. 5 and 6. Additionally, safe operating conditions include frequency ranges where only one stable solution is likely to occur, e.g. in this particular design between 4 and 6 Hz. Finally, the system could be equipped with an intelligent control system, where the restoring force or the damping through the coils could be changed in order to keep the magnet oscillating with a high, stable amplitude. However, it should also be remarked that in experimental systems the fully chaotic phase space found here will tend to collapse to a few steady state solutions because most typically friction will make the very narrow phase space branches unstable.

It is also worth mentioning that for non-linear dynamical systems such as the one investigated here, many of the simplifications normally satisfied by linear systems do not apply. In particular, the resulting motion depends on the amplitude of the driving oscillations in a non-trivial way. Moreover, the superposition principle is not applicable. The lack of this property means that when the driving oscillation is described by a superposition of oscillations with different frequencies, the resulting oscillation of the levitated magnet is not necessarily given by a superposition of the solutions individually associated with each of the driving frequencies.

As shown in the results section, the power produced by the investigated device is on the order of mW. While this is enough to power certain Internet-of-Things (IoT) device, it is still worth remarking that the output power of the investigated device is not optimized in this study, as it is the dynamics of the harvester that is instead investigated. The output power could potentially be improved by in a number of ways. The most straightforward improvements would be larger and/or more coils and stronger magnets in the harvester. However, we also remark that the geometry of the device is not optimized and different dimensions of e.g. the length of the tube could potentially lead to a larger power being harvested.

## Conclusion

Here we have investigated the dynamics of a floating magnet vibration energy harvester device with a nonlinear restoring force and a position-dependent coil damping force using a numerical model. Experimentally verified expressions for the magnet-magnet interactions and magnet-coil interaction were used in the model for the force expressions. Moreover, the effect of external vibrations is included as a periodic motion of the whole device rather than being approximated by a predefined periodic driving force with constant amplitude acting on the floating magnet.

We show that the EMVEH state space exhibits features that are typical for chaotic dynamical systems. However, the state is more complicated than for the Duffing equation, which have previously been used to approximate EMVEH systems. We also showed that for a given frequency, there exists more than the two states known from traditional frequency response experiments. These additional states mostly have a induced power between the known high power and low power states, but states can exists with a higher induced power than these, as was specifically seen in the EMVEH subjected to gravity studied here.

The consequences of approximating the restoring magnetic force by a polynomial function of increasing order was also investigated. It was shown that at high frequencies a high polynomial order is needed to accurately predict the motion of the floating magnet.

## Supplementary information

Supplementary video 1

## Data Availability

All data shown in this manuscript are available at Ref.^32^. A movie showing the phase space as function of frequency is available as supplementary material.
